# Gel immersion method for acute hemorrhagic rectal ulcer provides effective hemostasis without manual disimpaction or position changes

**DOI:** 10.1055/a-2471-8153

**Published:** 2024-12-10

**Authors:** Daisuke Orita, Yoshihiro Okabe, Takahiro Oribe, Mizuka Yonezawa, Yuichi Hirata, Michitaka Kohashi, Takuya Mimura

**Affiliations:** 1 Department of Gastroenterology, Kakogawa City Central Hospital, Kakogawa, Japan

Acute hemorrhagic rectal ulcer (AHRU) is characterized by sudden, painless, and significant rectal bleeding, often observed in patients with severe comorbidities 1 . Endoscopic hemostasis for AHRU is often challenging due to poor visibility caused by blood clots and fecal masses. Consequently, frequent manual disimpaction and position changes are often required to improve visibility, delaying the identification of the bleeding source and increasing patient discomfort. Studies show that using a high-viscosity gel (Viscoclear; Otsuka Pharmaceutical Factory, Tokushima, Japan) during emergency endoscopy for gastrointestinal hemorrhage improves visibility and helps identify the bleeding site 2 3 . When introduced into the rectum, the gel displaces both fecal material and blood clots toward the proximal end, causing them to float within the gel, which exposes hidden ulcers and the bleeding source ( Fig. 1 ). This report demonstrates that the gel immersion technique is effective in managing both active and inactive bleeding in AHRU cases ( Video 1 ).

**Fig. 1:**
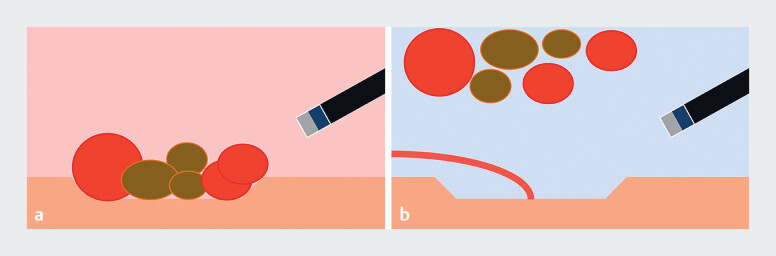
Schematic of the gel immersion method for acute hemorrhagic rectal ulcer. **a** Before applying the gel immersion method. **b** After applying the gel immersion method, bloody clots and fecal masses float within the gel, exposing the ulcer.

The gel immersion method for acute hemorrhagic rectal ulcer improved the visual field and allowed hemostasis to be achieved without the need for manual disimpaction or position changes.Video 1

In a case of active bleeding, a 93-year-old woman with a femoral neck fracture underwent emergency endoscopy. Visibility was initially compromised by blood clots and fecal masses ( Fig. 2 **a** ). Upon injection of the gel, both blood clots and fecal masses floated within the gel, significantly improving visibility ( Fig. 2 **b** ). Notably, blood clots attached to the rectal wall could be peeled off with the endoscope, allowing the clot to float freely in the gel.

**Fig. 2:**
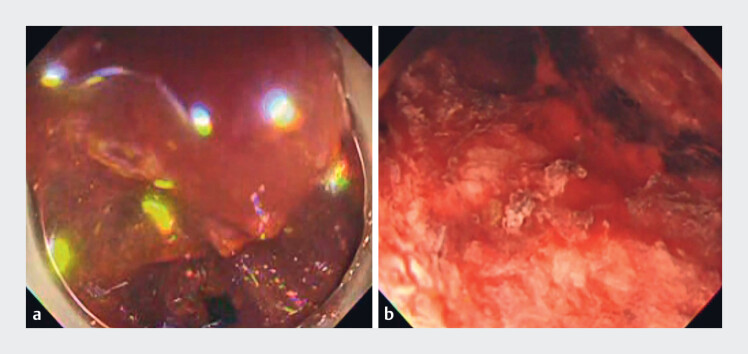
Comparison of endoscopic images before and after the gel immersion method. **a** Before the gel immersion method, the field of view was poor due to fecal masses and blood clots. **b** The gel immersion method provided a good field of view, and the bleeding point could be visualized.

In another case of inactive bleeding, a 98-year-old woman with bacteremia benefited from the gel immersion method during her endoscopic procedure. The technique facilitated the identification of small vessels within the ulcer base ( Fig. 3 ).

**Fig. 3:**
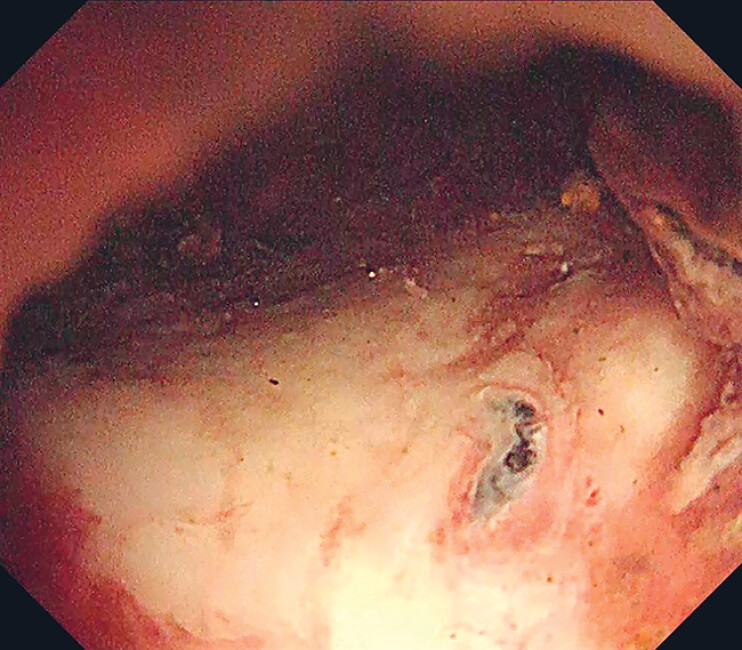
The good visibility allowed identification of even a small, exposed blood vessel at the base of the ulcer.

In both cases, manual disimpaction and position changes were unnecessary. Hemostasis was successfully achieved using electrocoagulation under gel immersion ( Fig. 4 ). This demonstrates that the gel immersion technique in AHRU management allows for the rapid identification of bleeding sites and is expected to reduce the procedure time.

**Fig. 4:**
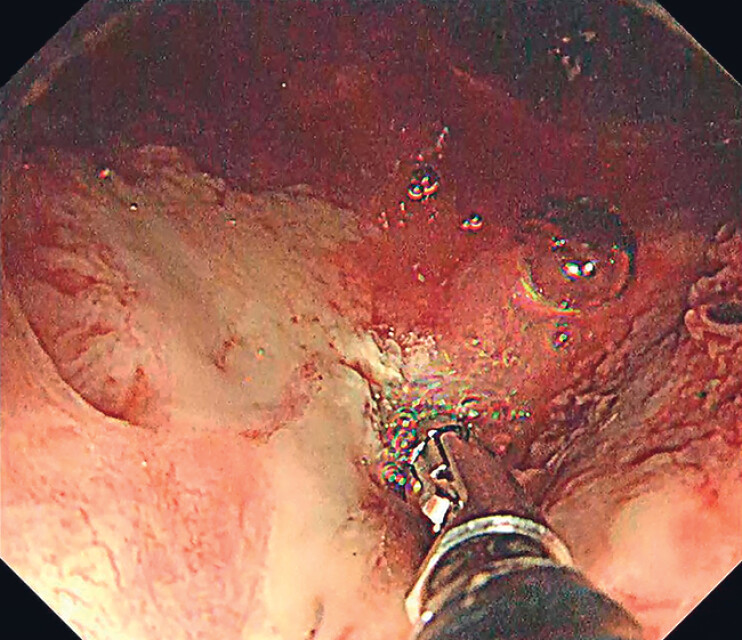
Endoscopic image of hemostasis by electrocoagulation under gel immersion.

Endoscopy_UCTN_Code_TTT_1AQ_2AZ

## Notes

Endoscopy E-Videos https://eref.thieme.de/e-videos
*E-Videos* is an open access online section of the journal *Endoscopy* , reporting on interesting cases and new techniques in gastroenterological endoscopy. All papers include a high-quality video and are published with a Creative Commons CC-BY license. Endoscopy E-Videos qualify for HINARI discounts and waivers and eligibility is automatically checked during the submission process. We grant 100% waivers to articles whose corresponding authors are based in Group A countries and 50% waivers to those who are based in Group B countries as classified by Research4Life (see: https://www.research4life.org/access/eligibility/ ). This section has its own submission website at https://mc.manuscriptcentral.com/e-videos .

## References

[1] Soeno T Shoji S Sakuraba K Acute hemorrhagic rectal ulcer accompanied with the brain disease Akita J Med 1981 8 207 213

[2] Yano T Nemoto D Ono K Gel immersion endoscopy: a novel method to secure the visual field during endoscopy in bleeding patients (with videos) Gastrointest Endosc 2016 83 809 811 26463338 10.1016/j.gie.2015.09.048

[3] Yano T Takezawa T Hashimoto A Gel immersion endoscopy: innovation in securing the visual field – clinical experience with 265 consecutive procedures Endosc Int Open 2021 9 E1123 E1127 34222638 10.1055/a-1400-8289 PMC8216780

